# Addition of low concentration of cholesterol-loaded cyclodextrin (CLC) has a positive effect on cryopreserved canine spermatozoa evaluated by andrological and biophysical methods

**DOI:** 10.1186/s12917-023-03851-6

**Published:** 2024-01-03

**Authors:** Zuzanna Ligocka, Agnieszka Partyka, Dorota Bonarska-Kujawa, Anna Mucha, Wojciech Niżański

**Affiliations:** 1 Department of Reproduction and Clinic of Farm Animals, Faculty of Veterinary Medicine, Wroclaw University of Environmental and Life Sciences, Plac Grunwaldzki 49, 50-366 Wroclaw, Poland; 2 Department of Physics and Biophysics, Faculty of Life Sciences and Technology, Wroclaw University of Environmental and Life Sciences, Norwida 25, 50-375 Wroclaw, Poland; 3 Department of Genetics, Faculty of Biology and Animal Science, Wroclaw University of Environmental and Life Sciences, Kożuchowska 7, 51-631 Wroclaw, Poland

**Keywords:** Spermatozoa, Cryopreservation, CLC, HBCD, Membrane fluidity, Cholesterol, Anisotropy, Generalized membrane polarization

## Abstract

**Background:**

This study was conducted to find the best concentration of cholesterol-loaded cyclodextrin (CLC) which has a positive impact on canine post thaw semen quality. Three different concentrations of CLC (0.83 mg/ml; 1.66 mg/ml; 3.32 mg/ml) and 2-hydroxylpropyl-beta-cyclodextrin (HBCD) (1.66 mg/ml) were used in addition to cryopreservation extender and compared with the control after thawing. Samples were assessed using computer-assisted semen analyzer (CASA), flow cytometry, fluorimeter by measuring the fluorescence anisotropy (ANISO) and determining the generalized membrane polarization (GP).

**Results:**

An addition of 0.83 mg/ml CLC significantly increased the percentage of progressive motile (PROG) and rapid spermatozoa (RAP) (*P* < 0.05). 1.66 mg/ml HBCD decreased progressive motility of spermatozoa and population with rapid movement relative to the control (*P* < 0.05). Furthermore, the groups with an addition of 1.66 mg/ml and 3.32 mg/ml of CLC, as well as the group with only cyclodextrin, increased percentage of dead spermatozoa without lipid peroxidation and decreased percentage of viable spermatozoa without LPO which was lower in these groups than in the control (*P* < 0.05). Other sperm parameters assessed on flow cytometer were not significantly different. The addition of CLC at 0.83 mg/ml and 3.32 mg/ml concentrations and 1.66 mg/ml of HBCD caused an increase in ANISO measured at 23 ºC (*P* < 0.05).

**Conclusions:**

In conclusion, the results suggest that increasing cholesterol in the plasma membrane of canine spermatozoa can improve their freezability. However, only low concentrations of CLC may improve semen quality after thawing without adversely affecting other parameters.

**Supplementary Information:**

The online version contains supplementary material available at 10.1186/s12917-023-03851-6.

## Background

Semen cryopreservation is widely used in many animal species. It allows for the storage of genetic material of highly valuable sires as well as animals in danger of xtinction [1, 2]. Among dog breeders, the freezing, banking, and shipping of semen is growing in interest. Many reproductive clinics have seen a significant increase in the processed shipments of frozen semen, especially with the onset of the COVID-19 pandemic. At the time when free movement was greatly restricted or even impossible, this type of service turned out to be the only way to carry out breeding plans.

Dogs are an animal species that share an environment with humans; thus, they are exposed to the same factors that cause a reduction in fresh and cryopreserved semen quality. For this reason, new freezing methods or additives are constantly being searched to ensure the least possible loss of semen quality during cryopreservation [3–5]. Many factors are known to affect the freezing result, such as age of male, physical conditions, and species sensitivity of sperm cell’s plasma membrane to phase changes during freezing from liquid to solid state and back to liquid state during thawing. Canine semen has a low resistance to cooling, similar to sperm from swine [6, 7]. The fluidity of a cell membrane has an immense impact on changes that take place in cells during freezing and at the time of thawing. Therefore, significant damage is done to the plasma membranes during fast and slow freezing [8]. In the case of cryopreserving canine male gametes, slow freezing is commonly used. However, this freezing method is burdened with a risk of solution effect. When dehydration of a cell occurs, it leads to rigidification of the membrane structure consisting of lipids and proteins. During thawing, the rigid structure of the membrane is disturbed, this is the key issue in development of spermatozoa damages during the freezing–thawing process [8].

Under physiological conditions, the cell membrane is in the liquid crystal phase. Changes in the conditions, including temperature, result in changes of the membrane fluidity and arrangement of phospholipid bilayer. The integrity of the cell membrane, and thus the viability and the vital functions of the cell, depend on many physico-chemical factors such as: temperature, pressure, osmotic pressure and the protein-lipid composition of the membrane [9]. Cholesterol which is a hydrophobic component of the cell membrane that plays an important role in modulation its structure. Fluidity, resistance to temperature changes and ion permeability of the lipid bilayer largely depend on the cholesterol content in the membrane. The high content of cholesterol in the membrane structure reduces the fluidity, increases the resistance to temperature changes and causes the formation of microregions with a greater order in the membrane. Lower cholesterol content causes greater fluidity and less order in the membrane structure [9, 10]. However, efflux of cholesterol is essential for sperm to undergo capacitation and to achieve fertilizing ability. On the other hand, supplementation of exogenous cholesterol to the plasma membrane has shown to mitigate the effects of cryocapacitation [11, 12]. Cholesterol management is therefore crucial in the storage of sperm so that, after thawing, the sperm can perform its vital functions and be able to fertilize.

Cholesterol can be added to or removed from the cell membrane by interaction with cyclodextrin. Cholesterol can be incorporated into the hydrophobic center of β-cyclodextrins forming cholesterol loaded cyclodextrin complex (CLC). Cyclodextrins are cyclic oligosaccharide sugars that are capable of encapsulating hydrophobic compounds such as cholesterol and transporting them to the cell plasma membrane [13, 14]. On the other hand cyclodextrins alone have a high affinity for sterols and can preferentially bind to cholesterol molecules in less ordered areas of the cell membrane, causing removal of sperm plasma membrane cholesterol and promoting mammalian sperm capacitation [15]. Moreover, the CLC complex is more soluble in the aqueous medium, and the incorporation of cholesterol into the membrane protects the cell membranes from damage caused by cell freezing. The CLC complex also exhibits antioxidant properties, and the cyclodextrin itself is also eager to complex the hydrophobic tails of other membrane phospholipids [16–19]. CLCs are assumed to prevent the rearrangement of phospholipids by cholesterol addition and increase membrane fluidity at low temperatures [20]. In other mammals, it has been found that the increased cholesterol content in the cell membranes of sperm positively affects their fluidity and, as a result, the quality of semen after thawing [11, 20–26]. Generally, treatment with variable range of the CLC (1–5 mg of CLC per 120 × 10^6^ of sperm) was proposed to be suitable to obtain remarkable improvements in the characteristics of sperm related to fertilization ability [13, 14, 21]. Therefore, the aim of the present study was to investigate the effect of low concentrations of CLCs on canine post thaw semen quality based on different analyses and evaluate their impact on different levels (kinematic, structural, functional and biophysical properties of spermatozoa). The 2-hydroxylpropyl-beta-cyclodextrin (HBCD) was used as a negative control. In this study we focused on motility of spermatozoa, structural and functional features and the research was extended to include analysis of biophysical properties of spermatozoa. The anisotropy (ANISO) and generalized polarization (GP) study has not yet been conducted on canine sperm. The impact of modification of canine spermatozoa cell membrane fluidity by altering the membrane cholesterol content was assessed to improve the efficiency of canine male gamete cells freezing protocol. Research on refining the process of male gametes cryopreservation is crucial in times of growing environment pollution, inbreeding and spread of infectious diseases negatively affecting canine semen quality which is constantly decreasing.

## Results

The sperm motility parameters of cryopreserved canine semen with HBCD and different concentrations of CLC are shown in Table 1. The percentage of sperm motility after thawing was more than 40% in the control and in the medium containing 1.66 mg/ml and 3.32 mg/ml of CLC. Additionally, in the medium containing 0.83 mg/ml of CLC motility after thawing was higher than 50%. Addition of 0.83 mg/ml CLC increased the percentage of progressive motile and rapid spermatozoa (*P* < 0.05) in comparison to the control. 1.66 mg/ml HBCD decreased progressive motility of spermatozoa and population with rapid movement relative to the control (*P* < 0.05) (Table 1).

**Table 1:** The effect of HBCD and CLC on the sperm motility parameters of frozen-thawed canine semen compared with the control group (*n* = 11). All data are presented as mean ± sd. Given *p*-value is for repeated measures ANOVA (A) or for Friedman test (F). Groups that differed statistically significantly from controls are marked with * (*p*-value < 0.05)

	**CONTROL**	**0.83 mg/ml CLC**	**1.66 mg/ml CLC**	**3.32 mg/ml CLC**	**1.66 mg/ml HBCD**	***p*-value**
MOT (%)	46.1 ± 17.7	57.5 ± 11.4	45.4 ± 13.8	48.1 ± 10.2	33.4 ± 10.3	0.0549 (F)
PMOT (%)	21.7 ± 11.9	31.2 ± 14.1*	22.7 ± 11.5	24.3 ± 11.3	13.6 ± 11.7*	0.0008 (A)
RAP (%)	24.2 ± 13.9	34.4 ± 16.2*	24.7 ± 13.2	27.3 ± 13.5	14.9 ± 13.2*	0.001 (A)
SLOW (%)	17.1 ± 10.3	19.5 ± 8.7	18.3 ± 8.4	17.4 ± 7.6	15.7 ± 6.5	0.198 (F)
ELONG (%)	60.4 ± 2.2	60.2 ± 1.7	59.9 ± 1.7	59.7 ± 1.8	59.8 ± 2.6	0.651 (F)
STR (%)	123.4 ± 20.1	128.6 ± 18.2	122.2 ± 14.6	126.3 ± 15.5	109.6 ± 24.5	0.051 (A)
LIN (%)	110.8 ± 16.2	116.6 ± 16.3	110.5 ± 11.7	113.1 ± 12.9	100.7 ± 22.0	0.101 (A)
VAP (µm/s)	177.6 ± 30.3	179.8 ± 26.4	174.2 ± 25.6	182.4 ± 24.0	152.0 ± 38.8	0.028 (A)
VSL (µm/s)	6.8 ± 0.8	6.7 ± 0.9	6.7 ± 0.9	7.0 ± 0.7	6.2 ± 0.9	0.0825 (F)
VCL (µm/s)	26.8 ± 3.9	27.1 ± 4.2	6.7 ± 0.9	26.8 ± 3.8	25.5 ± 4.1	0.688 (A)
ALH (µm/s)	88.5 ± 1.8	89.3 ± 2.4	89.1 ± 2.3	88.0 ± 2.2	90.3 ± 1.9	0.099 (A)
BCF (Hz)	63.1 ± 3.2	65.1 ± 5.4	64.5 ± 5.6	62.6 ± 4.2	66.8 ± 4.6	0.144 (A)

(MOT) the percentage of motile sperm, (PMOT) the percentage of progressively motile spermatozoa, (RAP) percentage of rapid spermatozoa, (SLOW) percentage of slow spermatozoa, (ELONG) elongation, (STR) straightness, (LIN) linearity, (VAP) path velocity, (VSL) progressive velocity, (VCL) curvilinear line velocity, (ALH) amplitude of lateral head displacement, (BCF) beat cross frequency.

The sperm characteristics evaluated by flow cytometry are shown in Tables 2 and 3. The addition of 1.66 mg/ml and 3.32 mg/ml of CLC increased the population of dead spermatozoa without LPO and decreased the population of viable spermatozoa without LPO compared with the control group (*P* < 0.05). The same effect was observed when 1.66 mg/ml HBCD was used. The population of viable spermatozoa without LPO was decreased in comparison to the control (*P* < 0.05). Using the concentration of 1.66 mg/ml of HBCD exhibited increasing dead spermatozoa with LPO compared to the control (*P* < 0.05) (Table 2). The other parameters eg. Sperm membrane integrity, sperm mitochondrial activity, sperm acrosome status, apoptosis and membrane lipid disorder and chromatin status had no significant differences compared to the control (Table 3). The sperm membrane integrity and mitochondrial activity showed *p*-value indicating statistically significant differences but there were no differences in comparison to the control group (Table 3).

**Table 2:** The effect of HBCD and CLC on the lipid peroxidation (C11-BODIPY581/59/PI) in the canine cryopreserved sperm compared with the control group (*n* = 11). All data are presented as mean ± sd. Given *p*-value is for repeated measures ANOVA (A) or for Friedman test (F). Groups that differed statistically significantly from controls are marked with * (*p*-value < 0.05)

	**CONTROL**	**0.83 mg/ml CLC**	**1.66 mg/ml CLC**	**3.32 mg/ml CLC**	**1.66 mg/ml HBCD**	***p*-value**
DEAD WITHOUT LPO (%)	55.12 ± 7.94	57.27 ± 8.16	64.20 ± 11.93*	63.54 ± 10.04*	62.70 ± 7.19*	0.027 (A)
DEAD WITH LPO (%)	1.34 ± 0.58	1.47 ± 0.51	1.67 ± 0.81	1.65 ± 0.94	1.94 ± 1.04	0.314 (A)
LIVE WITHOUT LPO (%)	42.50 ± 7.82	40.27 ± 8.16	33.36 ± 11.39*	34.13 ± 10.23*	34.37 ± 6.59*	0.024 (A)
LIVE WITH LPO (%)	1.04 ± 0.56	0.99 ± 0.68	0.77 ± 0.82	0.67 ± 0.38	0.99 ± 0.77	0.0351 (F)

**Table 3:** The effect of HBCD and CLC on the sperm membrane integrity (SYBR-14/PI), sperm acrosome status (PNA), sperm mitochondrial activity (JC-1/PI), apoptosis and membrane lipid disorder (YO-PRO-1/M540) and sperm chromatin structure status (AO) in the canine cryopreserved sperm compared with the control group (*n* = 11). All data are presented as mean ± sd. Given *p*-value is for repeated measures ANOVA (A) or for Friedman test (F). Groups that differed statistically significantly from controls are marked with * (*p*-value < 0.05)

	**CONTROL**	**0.83 mg/ml CLC**	**1.66 mg/ml CLC**	**3.32 mg/ml CLC**	**1.66 mg/ml HBCD**	***p*-value**
LIVE (%)	51.24 ± 18.27	51.82 ± 13.17	33.50 ± 11.67	42.34 ± 12.82	37.39 ± 15.59	0.005 (A)
L/IACR (%)	36.51 ± 9.48	40.09 ± 8.49	33.84 ± 10.60	33.16 ± 8.51	33.67 ± 9.70	0.232 (A)
HMMP (%)	55.98 ± 16.04	65.19 ± 12.91	56.55 ± 18.44	46.95 ± 14.17	50.09 ± 16.39	0.00163 (F)
L/STPM (%)	39.29 ± 7.24	43.46 ± 9.62	40.30 ± 8.79	36.41 ± 8.04	34.01 ± 10.92	0.056 (A)
DFI (%)	3.57 ± 0.77	3.58 ± 1.18	4.55 ± 1.44	4.53 ± 2.10	4.20 ± 0.66	0.650 (F)

The Table 4 shows results of anisotropy (ANISO) and generalized polarization (GP) measurement of sperm membrane, at the room temperature (23 ºC) and body temperature (38 ºC). In all groups ANISO at 38 ºC practically was not changed with respect to the control (*P* < 0.05). At the temperature 23 ºC an increase in the fluorescence anisotropy in relation to the control was shown. The addition of CLC in 0.83 mg/ml and 3.32 mg/ml concentrations and 1.66 mg/ml of HBCD caused an increase in ANISO compared with the control group in RT (Table 4). Analysis of GP at the temperature 23 ºC showed statistically significant differences but there were no differences in comparison to the control group (Table 4).

**Table 4:** The effect of HBCD and CLC on the anisotropy (ANISO) and general polarization (GP) of cryopreserved canine sperm plasma membranes compared with the control group (*n* = 11). All data are presented as mean ± sd. Given *p*-value is for repeated measures ANOVA (A) or for Friedman test (F). Groups that differed statistically significantly from controls are marked with * (*p*-value < 0.05)

	**CONTROL**	**0.83 mg/ml CLC**	**1.66 mg/ml CLC**	**3.32 mg/ml CLC**	**1.66 mg/ml HBCD**	***p*-value**
ANISO 23 °C	0.109 ± 0.003	0.122 ± 0.014*	0.111 ± 0.003	0.123 ± 0.017*	0.115 ± 0.007*	0.00108 (F)
ANISO 38 °C	0.079 ± 0.004	0.081 ± 0.004	0.082 ± 0.004	0.081 ± 0.005	0.081 ± 0.004	0.784 (F)
GP 23 °C	0.460 ± 0.027	0.478 ± 0.023	0.457 ± 0.017	0.474 ± 0.018	0.483 ± 0.021	0.025 (A)
GP 38 °C	0.455 ± 0.030	0.462 ± 0.037	0.456 ± 0.035	0.477 ± 0.031	0.480 ± 0.027	0.0509 (F)

The results of the analysis of the correlation between the examined structural and functional parameters of semen in the group 0.83 mg/ml CLC (upper triangle) and in the control group (lower triangle) are presented in Table 5. The strongest positive, statistically significant correlation was shown in the group with 0.83 mg/ml CLC, between the percentage of sperm with detectable DNA fragmentation (DFI) and the population of spermatozoa with slow movement (0.89). A similar positive correlation was noted between the population of spermatozoa with high mitochondrial potential (HMP) and percentage of spermatozoa with progressive movement and rapid sperm population, where the value of correlation coefficients was 0.72 and 0.71, respectively. The strongest negative, statistically significant correlation was found between the spermatozoa with HMP and population of slow spermatozoa with the value -0.78.

**Table 5:** Pearson’s correlation matrix of cryopreserved semen features for the group 0.83 mg/ml CLC (upper triangle) and for the control group (lower triangle)

	**LIVE WITHOUT LPO**	**HMMP**	**L/IACR**	**DFI**	**L/STPM**	**LIVE**	**MOT**	**PMOT**	**RAP**	**SLOW**
LIVE WITHOUT LPO	**0.57**	0.00	-0.14	-0.04	0.19	0.14	-0.22	-0.22	-0.21	-0.05
HMMP	-0.37	**0.31**	0.31	-0.68*	0.66*	-0.46	0.06	0.72*	0.71*	-0.78*
L/IACR	0.19	0.14	**0.14**	-0.23	0.60	0.05	0.78*	0.55	0.57	-0.12
DFI	-0.16	-0.52	-0.48	**0.22**	-0.39	0.72*	0.08	-0.79*	-0.81*	0.89**^b^
L/STPM	0.75*	0.11	0.12	-0.35	**-0.34**	0.18	0.35	0.61*	0.59	-0.39
LIVE	0.52	-0.36	0.54	0.01	0.43	**0.38**	0.13	-0.52	-0.54	0.69*
MOT	-0.08	0.36	0.67*	-0.43	0.11	0.46	**-0.10**	0.32	0.32	0.26
PMOT	-0.53	0.69*	0.41	-0.39	-0.32	-0.27	0.48	0.61*	1.00***	-0.78*
RAP	-0.56	0.70*	0.35	-0.37	-0.34	-0.33	0.42	1.00***	0.67*	-0.79*
SLOW	0.20	-0.13	0.30	-0.12^a^	0.27	0.62*	0.73*	-0.22	-0.29	0.65*

Coefficients marked with *, **, *** were statistically significant (*p*-value < 0.05, 0.01, 0.001, respectively). Corresponding correlation coefficients marked with different letters were statistically significantly different

**Fig. 1:**
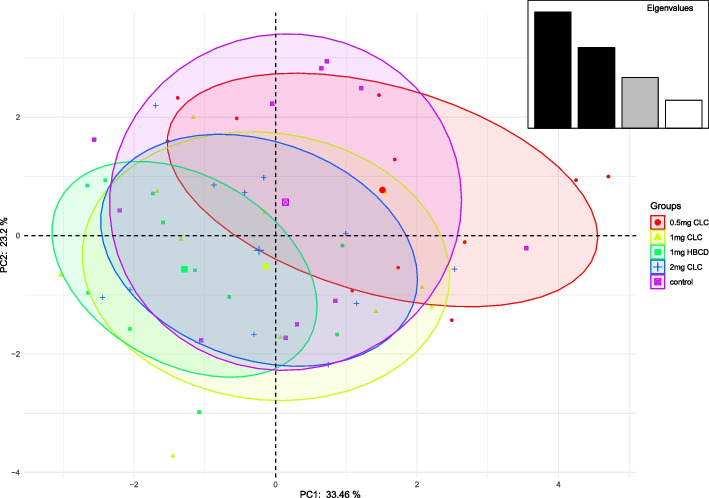
PCA scatter plot for the 1st and 2nd principal component analysis for the features in Table 6

**Fig. 2:**
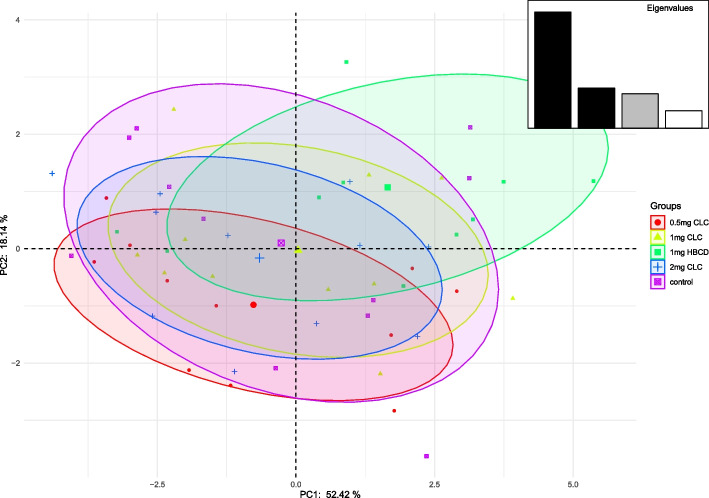
PCA scatter plot for the 1st and 2nd principal component analysis for the features in Table 7

Principal component analysis (PCA) results for selected parameters of spermatozoa are shown in Figs. 1 and 2. There was noticed low separation of the control and the experimental groups. The highest separation was shown between groups with 0.83 mg/ml CLC and 1.66 mg/ml HBCD. For the PCA analysis (Tab. 6, 7), the first two principal components (PC1, PC 2) were considered according to the rule that their eigenvalues should exceed 1 and the total should explain more than 75% of the variation. In this case, however, it was not reasonable to consider the other components (PC3 and PC4), since they were not strongly related to the analyzed characteristics. Principal component analysis of PC1 and PC2 (Table 6) showed the highest differences in progressive and rapid spermatozoa between the group with 0.83 mg/ml of CLC and 1.66 mg/ml of HBCD. They showed that the group with addition 0.83 mg/ml of CLC had a larger population of spermatozoa with HMP, rapid movement and higher percentage of progressive and live spermatozoa than the group with addition of 1.66 mg/ml of HBCD (Table 6, Fig. 1). Principal component analysis of PC1 and PC2 (Table 7) showed the highest differences in progressive and rapid spermatozoa between group with 0.83 mg/ml of CLC and 1.66 mg/ml of HBCD, 1.66 mg/ml of CLC and 3.32 mg/ml of CLC (Table 7, Fig. 2).

**Table 6:** Eigenvalues, percent of cumulative explained variance and loadings of first four principal components of PCA of frozen-thawed dog semen parameters

	**PC1**	**PC2**	**PC3**	**PC4**
**Eigenvalue**	3.35	2.32	1.46	0.80
**Cumulative variance (%)**	33.46	56.67	71.28	79.29
LIVE WITHOUT LPO	-0.05	0.60	-0.37	0.50
HMMP	0.73	-0.21	-0.02	-0.24
L/IACR	0.52	0.57	-0.30	0.10
DFI	-0.48	-0.07	0.60	-0.03
L/STPM	0.48	0.26	-0.35	-0.50
LIVE	0.15	0.81	-0.02	-0.15
MOT	0.59	0.47	0.62	0.08
PMOT	0.91	-0.24	0.22	0.16
RAP	0.90	-0.27	0.21	0.18
SLOW	-0.28	0.72	0.51	-0.14

**Table 7:** Eigenvalues, percent of cumulative explained variance and loadings of first four principal components of PCA of frozen-thawed dog semen parameters

	**PC1**	**PC2**	**PC3**	**PC4**	**PC5**
**Eigenvalue**	6.29	2.18	1.86	0.94	0.31
**Cumulative variance (%)**	52.42	70.56	86.04	93.92	96.54
MOT	-0.26	-0.86	0.03	-0.41	0.01
PMOT	-0.88	-0.30	-0.25	-0.22	0.05
RAP	-0.89	-0.26	-0.22	-0.23	0.06
SLOW	0.60	-0.70	0.23	-0.13	-0.08
VAP	-0.91	-0.17	-0.24	0.26	0.04
VSL	-0.84	-0.21	-0.36	0.31	0.03
VCL	-0.95	-0.09	0.06	0.27	0.03
ALH	-0.77	0.15	0.53	0.20	0.07
BCF	0.30	-0.71	0.23	0.49	-0.26
STR	0.65	-0.11	-0.63	0.29	0.02
LIN	0.52	-0.07	-0.83	0.00	0.04
ELONG	-0.70	0.40	-0.27	-0.22	-0.47

## Discussion

In recent years, the freezing, storing, and transporting of canine semen is a growing need. Moreover, freezing and global shipping of the semen enables the gene pool exchange in dog kennels. In fact, during the COVID-19 pandemic, only these procedures allowed dog breeders to carry out their breeding plans, even when even travel to the neighboring countries was difficult or impossible. Dogs can be classified as “good freezers” and “bad freezers” because there are known individual cases, despite good quality of raw semen, the parameters motility, abnormal morphology and abnormal acrosomes were significantly changed after thawing [27, 28]. Considering also short lifespan spermatozoa after thawing in combination with long periods of the active phase of the cycle in bitches, the insemination day must be calculate with high precison. Thus whelping rates when cryopreserved sperm were used still are not satisfied [29]. Therefore, investigation of new extenders and protocols for cryopreservation is still needed.

In the present study we compared three concentrations of CLC 0.83, 1.66, 3.32 mg/ml (0.5; 1.0; 2.0 mg per 120 × 10^6^ sperm) and 1.66 mg/ml (1.0 mg per 120 × 10^6^ sperm) of 2-hydroxypropyl-β-cyclodextrin (HBCD) as a negative control. Regarding motility parameters in other papers [8, 21, 30] our results confirm that the lowest CLC concentration has a positive impact on freezing dog semen. We found that the post-thawed sperm motility in the control and groups with CLC was very high compared to the motility obtained by other authors using similar cryopreservation procedures where the motility ranged from about 23% to 61% [31, 32]. Based on the results of sperm motility and progressive motility in the control group, the semen samples collected from beagle males can be classified as a”good freezers’’ [27, 33, 34]. Results from “bad freezers” with the addition of CLC in future studies may reveal worthwhile findings. In the studies performed on stallion semen the best MOT and PROG results were obtained with 1.5 mg/120 × 10^6^ sperm CLC addition. The significantly higher number of cells with progressive movement and rapid cells obtained with spermatozoa cryopreserved in the extender with 0.5 mg/120 × 10^6^ sperm of CLC confirmed the more effective preservation of sperm cell function exerted by cholesterol insertion to cell membrane. The best concentration of cholesterol loaded cyclodextrins was 0.5 mg/120 × 10^6^ sperm, and this result confirms similar findings in gazelle [35] and bull [36]. It has been reported that other low concentrations (0.75 mg/120 × 10^6^ sperm, 1 mg/120 × 10^6^ sperm and 1.5 mg/120 × 10^6^ sperm) of CLC had a positive effect on motility parameters in ram [22] and bull [21]. Whereas, we have found that even small concentrations of HBCD (2-hydroxypropyl-ß-cyclodextrin) significantly decreased MOT, PROG and rapid cells population, which corresponds to other authors [37].

Concentration of CLC which has beneficial influence on cryosurvival rates definitely depends on the sperm membrane cholesterol to phospholipid ratio [12, 38] which is specific for different species. The sperm from species having a relatively lesser cholesterol to phospholipid ratio (e.g., stallion, ram, and bull) are more prone to cold shock-induced damage than the sperm with a greater cholesterol to phospholipid ratio (e.g., rabbit and human) [39, 40]. It was also revealed that in the same species changes of concentration in seminal plasma cholesterol are reflected in fertility parameters of stud males in horses [41] and bovine [42, 43]. The same findings are observed in research on dogs. Dogs which are classified as a good freezers had significantly higher cholesterol concentration in seminal plasma than in the bad freezers group [27]. Taking into account the best CLC concentration as an addition to cryopreservation of dog semen, the age of the dog should also be a considered factor. It is proven that increasing age in dogs is associated with a decline in vitro spermatological parameters, such as motility, viability, mitochondrial activation, normal morphology, and DNA integrity. Better freezability characteristics were observed when dogs were young [44].

The integrity of sperm membranes is crucial to maintain spermatozoal functions during storage in the female’s reproductive tract and oocyte’s penetration [38]. In the present study, membrane integrity tests showed that groups with addition of 1 mg/120 × 10^6^ sperm and 2 mg/120 × 10^6^ sperm CLC and 1 mg/120 × 10^6^ sperm of HBCD had the lowest percentage of live spermatozoa. These results are contrary to studies performed on semen collected from 4 adult German Shepherds carried by Khan et al. [25]. In the mentioned publication, the ideal CLC concentration was 2 mg/120 × 10^6^ sperm which was proven by the motile, viable, plasma membrane intact, acrosomal intact and DNA intact spermatozoa results. We may see the reason for these contradictory results in the low semen quality of the German Shepherds in the control group. When we look at results in this group these dogs can be classified as a “bad freezers’’. In this case higher concentration of CLC may have been needed to obtain improvement of freezing results. Assessment of the sperm membrane’s integrity also includes acrosomal status as a fundamental in cryopreserved spermatozoa. Acrosomes take part in the penetration of oocyte’s zona pellucida and that means that acrosomal reaction must occur at the relevant time [45]. In the study of Khan et al. [25] the population with intact acrosome was significantly higher in the 2 mg/120 × 10^6^ sperm CLC group (*p* < 0.05). However, our study did not show significant differences in this parameter after CLC exposition.

It is known that high mitochondrial membrane potential is lower in the freeze-thawed semen than in the fresh semen. There are studies where some additives to the cryopreservation extenders increase the population of canine spermatozoa with HMMP after thawing [3]. Although in our study, the addition of CLC did not improve, and a strong positive correlation between the sperm with high mitochondrial potential and progressive and rapid sperm was found in the 0.5 mg/120 × 10^6^ sperm of CLC.

Sperm apoptosis and membrane lipid disorder were evaluated with the stains YO-PRO-1 and M540. The M540 stain increases its fluorescence when lipids are in a high state of disorder, and it detects changes in plasma membrane fluidity. Similar to other authors [46] this test didn’t show differences in lipid organization in our studied groups. Because of this, it was important to assess membrane fluidity by measuring the fluorescence anisotropy. Measurement of anisotropy and general polarization might therefore be used as better, more specific tests for evaluation lipid organization in plasma membranes of spermatozoa. The results obtained from ANISO and GP analysis will be discussed below.

Lipid peroxidation is a chain reaction which leads to formation of lipid peroxides and cytotoxic aldehydes [47]. It is known that reactive oxygen species (ROS) which are produced during the freezing procedure increase LPO in spermatozoa after freezing–thawing procedure [48]. The present results showed that all additives decreased the population of viable spermatozoa without LPO but only in the group with 0.5 mg/120 × 10^6^ sperm of CLC the decrease was not significant. It means that only low concentration of CLC did not exert a significantly negative effect on spermatozoa considering viability. But the highest concentration of CLC (2 mg/120 × 10^6^ sperm) had a significant negative influence on lipid peroxidation effect on spermatozoa during cryopreservation. Considering the fact that high levels of ROS cause destruction of the lipid matrix structure during freezing procedure, it is interesting that using CLC and HBCD to modify plasma membrane content did not cause negative changes in LPO [49].

It is well known that the integrity of the nuclear DNA during freezing–thawing procedure could be negatively affected. Although spermatozoa with DNA damage may be able to fertilize an oocyte, that could potentially disturb (epi)genetic regulation of the early embryo and block its further development [50]. In our study there were no differences in groups with CLC or HBCD compared to the control in opposition to *Khan *et al*.* [25] who observed that DNA intact spermatozoa were significantly higher in 2 mg/120 × 10^6^ sperm CLC group.

Even when we look at some features of spermatozoa separately there are no differences between the groups with additions compared to the control whereas we can observe interesting correlations using different statistical analysis (Pearson’s correlation and PCA). Some of these correlations are well known and reported before. For example, the positive correlation between the population of spermatozoa with high mitochondrial potential (HMP) and percentage of spermatozoa with progressive movement and rapid sperm population [51]. The negative correlation between the HMMP and population of slow spermatozoa movement observed in our study is not surprising. On the other hand, a positive correlation between the percentage of sperm with detectable DNA fragmentation (DFI) and the population of spermatozoa with slow movement was seen. Whereas PCA analysis confirms results obtained in previous tests and revealed the best concentration of CLC. It was shown that the group with addition 0.5 mg/120 × 10^6^ sperm of CLC has better results considering PROG, RAP, mitochondrial potential and live spermatozoa than the group with addition of 1 mg/120 × 10^6^ sperm of HBCD. In addition, progressive and rapid spermatozoa populations were higher also in the group with 0.5 mg/120 × 10^6^ sperm of CLC than 1 mg/120 × 10^6^ sperm of CLC and 2 mg/120 × 10^6^ sperm of CLC.

As mentioned above, in the present study, changes in the fluidity and general polarization (GP) of the cryopreserved sperm membranes were determined. The tests were performed at the room temperature of 23 °C and at the physiological temperature of 38 °C. We observed that the cell membrane is not completely liquid crystalline phase at room temperature and has gel domains, as other authors have noted [9, 10]. The addition of HBCD and CLC caused the stiffening of the membrane hydrophobic interior, as evidenced by higher ANISO values. This may indicate a slightly higher cholesterol content in the membranes of frozen sperm with additives. Treatment by HBCD and CLC did not change the fluidity of the membrane in the hydrophobic area in the body temperature, which may also indicate a similar cholesterol content in the tested and control samples. Therefore, studies indicate that cyclodextrin and its complexes under physiological conditions show their activity by interacting primarily with the hydrophilic surface of the membrane and significantly influencing its greater order [16–19].

Like most studies, this one has some limitations: regarding the animals, there was a low concentration of spermatozoa in ejaculate collected from one dog. It was not possible to divide ejaculate from one dog on 4 experimental groups and the control group and cryopreserve at least 2 straws for each group. This was the reason why we pooled semen and it let us obtain a unified semen sample.

## Conclusion

In conclusion, the results suggest that increasing cholesterol in the plasma membrane of canine spermatozoa can improve spermatological parameters, such as motility parameters (MOT, PROG, RAP) and plasma membrane integrity. Moreover 0.83 mg/ml of CLC prevents canine sperm against lipid peroxidation post thaw. The best freezability characteristics were observed when the 0.83 mg/ml of CLC was used.

## Methods

### Reagents

The fluorescent probes: Live/Dead Sperm Viability Kit: SYBR-14, propidium iodide (PI); PNA from Arachis hypogaea Alexa Fluor® 488 conjugate; JC-1; YO-PRO-1; Merocyanine 540, C_11_-BODIPY^581/591^; acridine orange (AO); 6-dodecanoyl-2-dimethylaminonaphthalene (Laurdan); and 1,6-diphenyl-1,3,5-hexatriene (DPH) were purchased from Thermo Fisher Scientific Inc., Waltham, MA, USA. Rest of chemicals were bought from Sigma-Aldrich Co., St. Louis, Missouri, USA.

### Extender

The Tris-citric acid–fructose–egg yolk extender (TFE) was composed of Tris (hydroxymethyl)- aminomethane (0.2 M), citric acid monohydrate (0.06 M), fructose (0.05 M), distilled water, and 20% (v/v) of egg yolk [1, 52]. This extender was used throughout the whole study and was supplemented with the stated concentrations of cyclodextrins and cyclodextrin-cholesterol complexes.

### Cyclodextrin and cyclodextrin-cholesterol complex (CLC) preparation

2-Hydroxypropyl-ß-cyclodextrin (HBCD) and cyclodextrin-cholesterol complex (CLC) were prepared according to the procedure adopted by Mocé et al. [22]*.* Initially, HBCD was dissolved in methanol in proportion 1 g of HBCD and 2 ml of methanol. The 200 mg of cholesterol was used to made solution with 1 ml of chloroform. In the next step, CLC was prepared using 0.45 ml aliquot of cholesterol dissolved in chloroform and was added to 2 ml of methanol with HBCD. Each mixture was stirred until the combined solution was clear. The solvent was evaporated using nitrogen gas and then the crystals of HBCD and CLC were allowed to dry in a desiccator. Then the reagents were stored at 22 ºC until use. The working solutions were prepared similarly, by adding 20 mg of crystals to 1 ml of Tris extender at 37 °C and stirring the solution using a vortex mixer.

### Animals

The experiment was carried out using five mature (3–6 years old) Beagle males from the kennel owned by the Department of Reproduction and Clinic of Farm Animals in Wroclaw University (reg. 0057). The animals were in good health and normal reproductive condition. They were fed dry food once daily, with free access to water. Differences caused by individual properties and age of dogs [1, 53, 54] were limited using pooled semen and dogs between 3 and 6 years old. Procedures performed in our experiments did not require 2nd Local Ethical Committee in Wroclaw approval (statement n. 025/2020).

### Semen collection and processing

The semen from each dog was collected by manual stimulation twice a week. The sperm-rich fraction of each ejaculate was collected into a calibrated collection tube with water-coat pre-warmed to 37 °C. Each ejaculate was analyzed to determine its sperm concentration, the total number of spermatozoa and sperm motility, to obtain an adequate semen quality. After these analyses, all ejaculates were pooled. The semen was collected 11 times from each dog.

### Cryopreservation and thawing method

The pool of semen was divided into five aliquots in order to evaluate 3 different concentrations of CLC, 1 concentration of HBCD as additions to cryopreservative extender (TFE): 1) HBCD 1 mg/ 120 × 10^6^ cells (1.6 mg/ml), 2) CLC 0.5 mg/120 × 10^6^ cells (0.83 mg/ml), 3) CLC 1 mg/120 × 10^6^ cells (1.6 mg/ml), 4) CLC 2 mg/120 × 10^6^ cells (3.3 mg/ml) and compare to the 5) control without any additions. The four treated samples were incubated for 15 min at 22 °C and then subjected to cryopreservation. To the control sample, an equivalent volume of Tris-based extender was added and sample was incubated before cryopreservation too. All samples were extended with TFE to a final concentration of 200 × 10^6^ spermatozoa/ml. Each extended semen was cooled to 5 °C over 1 h and then 6% (final concentration) of glycerol was added. Samples were equilibrated for 90 min at 5 °C. Straws were filled with the semen samples and then frozen on the horizontal rack at − 140 °C for 15 min with nitrogen vapor 5 cm above the liquid nitrogen and stored in liquid nitrogen [1, 52]. After 3 months of storage, the samples were thawed at 37 °C in a water bath for 60 s for detailed semen assessment.

### Post-thawed semen evaluation

#### CASA motility analysis

Sperm motility characteristics were evaluated using computer-assisted semen analyzer (CASA) Hamilton Thorne Sperm Analyser IVOS version 12.2 l (Hamilton Thorne Biosciences, MA, USA) under 1.89 × 10 magnification. Three µl aliquot of semen was placed in 20 µm Leja analysis chamber (Leja, Nieuw-Vannep, Netherlands) at 37 °C. The analysis was performed with the frame rate 60 Hz and five fields were randomly selected. The parameters measured were: the percentage of motile sperm (MOT), the percentage of progressively motile spermatozoa (PMOT), percentage of rapid spermatozoa (RAP), percentage of slow spermatozoa (SLOW), elongation (ELONG), straightness (STR), linearity (LIN), path velocity (VAP), progressive velocity (VSL), curvilinear line velocity (VCL), amplitude of lateral head displacement (ALH) and beat cross frequency (BCF).

#### Flow cytometric analyses

The analyses were performed on Guava EasyCyte 5 cytometer (Merck KGaA, Darmstadt, Germany). The fluorescent probes used in the experiment were excited by an Argon ion 488 nm laser. Acquisitions were done using the GuavaSoft™ 3.1.1 software (Merck KGaA, Darmstadt, Germany). A total of 40 mln of spermatozoa from all groups was suspended in 2.5 ml of tris based extender. In the next step every sample were divided to perform all tests. The non-sperm events were gated out based on scatter properties and not analyzed. A total of 10,000 events were analyzed for each sample.

Sperm membrane integrity was determined by a double-fluorescent labeling technique, according to the protocol described by Partyka et al. [55]. Briefly, 300 µL of the diluted samples were stained with 5 µL of SYBR-14 (commercial solution diluted 50-fold; 330 nM fianal solution) and 5 µL of 1.4 mM PI (propidium iodide). The PI negative and SYBR-14 positive population showing green fluorescence was considered alive, with the sperm plasma membrane intact (PMI), red-fluorescent sperm heads indicated dead spermatozoa, red and green-fluorescent sperm heads represent moribund (dying) spermatozoa.

Sperm acrosome status was assessed with lectin PNA from Arachis hypogaea Alexa Fluor® 488 conjugate. Diluted semen samples were mixed with 10 μL of PNA working solution (1 μg/mL; final concentration 0.02 µg/ml) and incubated for 5 min at room temperature (23 ºC) in the dark. After incubation, the samples were washed and 5 μL of PI were added before cytometric analysis [55]. Spermatozoa that were PI and PNA negative were considered as live with intact acrosomes (L/IACR).

Sperm mitochondrial activity was determined by staining with JC-1 and PI. A 3 mM stock solution of JC-1 in DMSO was prepared. From each sample, 500 μL of a sperm suspension containing 50 × 106 cells/mL was stained with 0.67 μL JC-1 (4 µM final solution) stock solution. The samples were incubated at 37 °C in the dark for 20 min before flow cytometric analysis. Spermatozoa emitting orange fluorescence were classified as having high mitochondrial membrane potential (HMMP) and those emitting only green fluorescence as having low mitochondrial membrane activity Partyka et al. [56].

Apoptosis and membrane lipid disorder were evaluated with the stains YO-PRO-1 (25 μM solution in DMSO) and M540 (1 mM solution in DMSO). Spermatozoa were diluted after thawing to 50 × 10^6^ cells/mL with tris based extender and 2.7 μL M540 (final concentration: 2.7 μM) and 1 μL of YO-PRO-1 (final concentration: 25 nM) were added to 1 mL of diluted spermatozoa [57]. Fluorescence was measured using a FL-2 sensor, a 575 nm band-pass filter to detect M540, and a FL-1 sensor and a 525 nm band-pass filter to detect YO-PRO-1. Cells with no YO-PRO-1 fluorescence and low M540 fluorescence were classified as live cells without apoptosis and a stable plasma membrane (L/STPM), and those with YO-PRO-1 fluorescence and high M540 fluorescence as apoptotic spermatozoa with an unstable plasma membrane (APOP/UNSPM).

The lipid peroxidation was evaluated using a fluorescent lipid probe C_11_-BODIPY^581/591^ as previously described [58]. Semen sample in volume 500 μL after thawing was centrifuged at 600 × g for 5 min to remove any Tris egg yolk extender and each sample was resuspended with Tris buffer. 1 μL of 2 mM C11-BODIPY581/591 in ethanol was added to the diluted samples (final concentration of 4 μM) and incubated for 30 min at 37 °C in the dark. The samples were subsequently centrifuged at 500 × g for 3 min and the pellet was resuspended in 500 μL of Tris buffer to remove any unbound dye probe or excessive probe. To determine the cell viability, the samples were stained with PI and further incubated for 5 min at room temperature before conducting cytometric analysis. Dot plots of C11-BODIPY581/59/PI stained spermatozoa showed four populations of cells: live spermatozoa without LPO (PI- BODIPYred), live spermatozoa with LPO (PI-BODIPYgreen), dead spermatozoa without LPO (PI_ BODIPYred) and dead spermatozoa with LPO (PI_ BODIPYgreen). Green fluorescence was measured using FL-1 and red fluorescence was measured using a FL-3 filter.

Acridine orange stain was used in the Sperm Chromatin Structure Assay (SCSA) with minor modifications of the previously described procedure [55]. The suspension (100 μL) was subjected to brief acid denaturation by mixing with 200 μL of lysis solution (Triton X-100 0.1% (v/v), NaCl 0.15 M, and HCl 0.08 M, pH 1.4), held for 30 s and there was mixing with 600 μL of AO solution [6 μg AO/mL buffer: citric acid (0.1 M), Na2HPO4 (0.2 M), EDTA (1 mM), NaCl (0.15 M), pH 6]. After 3 min, samples were analyzed for fluorescence. The sperm cell population with a normal doublestranded configuration of DNA had a green fluorescence (FL-1), which was considered to be the largest spermatozoa population. The cells, which had a greater amount of red fluorescence (FL-3), were located to the right of the main population indicating these cells had denatured DNA (DFI).

#### Membrane fluidity and generalized membrane polarization (GP) analyzes

Membrane fluidity and generalized membrane polarization (GP) analyses were used as a part of biophysical semen assessment. Membrane fluidity was assessed by measuring the fluorescence anisotropy (ANISO) with the fluorescent dye 1,6-diphenyl-1,3,5-hexatriene (DPH) inserted into the hydrophobic lipid fraction of the plasma membranes. The samples were suspended in 2 ml of Tris–EDTA diluent at a sperm concentration of 2 × 10^6^/mL with DPH working solution (2.5 M prepared from a DPH stock solution of 2 mM in dimethyl sulfoxide (DMSO)) in quartz cuvettes. The uptake of DPH did not increase the percentage of non-viable cells and did not affect sperm motility. The measurements were conducted with a fluorimeter (CARRY Eclipse, VARIAN, California, USA) at 23 °C (RT) and 38 °C. The excitation and emission wavelengths were λ_ex_ = 360 nm and λ_em_ = 425 nm, respectively. Fluorescence anisotropy (ANISO) for the DPH probe was calculated using the formula:

$$ANISO=\frac{({I}_{II} - {GI}_{\perp })}{( {I}_{II}+ {2GI}_{\perp })}$$

where I_II_ and I_┴_ = fluorescence intensities observed in directions parallel and perpendicular, respectively, to the polarization direction of the exciting wave. G is an apparatus constant dependent on the emission wavelength. Fluorescence anisotropy is a parameter indicative of changes in the fluidity of the membranes of HBCD and CLC induced sperm cells.

The arrangement of the hydrophilic part of the membrane was assessed on the basis of changes in the fluorescence intensity of the Laurdan probe (6-dodecanoyl-2-dimethylaminonaphthalene) by determining the generalized membrane polarization (GP), which was calculated from the formula [59]:

$$GP=\frac{({I}_{b} - {I}_{r})}{({I}_{b} + {I}_{r})}$$

where I_b_ = fluorescence intensity at λ = 440 nm, I_r_ = fluorescence intensity at λ = 490 nm. The samples were suspended in 2 ml of Tris–EDTA diluent at a sperm concentration of 2 × 10^6^/ml with Laurdan working solution (2.5 M prepared from a Laurdan stock solution of 2 mM in dimethyl sulphoxide (DMSO)) in quartz cuvettes. The measurements were conducted with the fluorimeter (CARRY Eclipse, VARIAN) at 23 °C and 38 ºC. The excitation wavelength was 360 nm, and the emitted fluorescence was recorded at two wavelengths, 440 and 490 nm.

### Statistical analysis

Straws from each group were thawed and obtained results were compared to the control.

The statistical analysis of the described research material was carried out with the use of the R 4.3.1 package [60].

The compliance of the distribution of the analyzed traits in the considered research groups with the normal distribution was verified by the Shapiro–Wilk test at the significance level α = 0.05. The homogeneity of the variability of traits in the groups was verified with the Bartlett test at the significance level α = 0.05.

The statistical significance of the influence of group on the values of the analyzed features was verified by the repeated measures ANOVA, if the necessary assumptions were met (compliance of the distribution with the normal distribution and homogeneity of variance), and by the Friedman test—otherwise. The post-hoc analysis included comparisons of the control group with the case groups and these were paired t test after the repeated measures ANOVA and paired Wilcoxon test after the Friedman test. The obtained *p*-values were corrected by the Benjamini–Hochberg method. The above part of the analysis was performed using the *rstatix* library [61].

Pearson's correlation analysis was performed using the *psych* library [62]. The statistical significance of the determined correlation coefficients was verified at the significance level of α = 0.05 with the FDR correction. The statistical significance of differences between the correlation coefficients determined within the groups under consideration (control or case) was verified on the basis of the confidence intervals constructed for these coefficients. If the confidence intervals of the corresponding coefficients did not overlap, these coefficients were considered statistically significantly different [63].

Basing on the available data, a principal component analysis (PCA) was also performed. This part of the analysis was carried out using the *ade4* [64–68] and *factoextra* [69] libraries.

### Supplementary Information

**Additional file 1: Figure S1.** Flow cytometric dot-plot distributions of spermatozoa and non-sperm particles after staining with SYBR-14 and PI. **Figure S2.** Flow cytometric dot-plot distributions of spermatozoa and non-sperm particles after staining with PNA and PI. **Figure S3.** Flow cytometric dot-plot distributions of spermatozoa and non-sperm particles after staining with JC-1 and PI. **Figure S4.** Flow cytometric dot-plot distributions of spermatozoa and non-sperm particles after staining with YO-PRO-1 and M540. **Figure S5.** Flow cytometric dot-plot distributions of spermatozoa and non-sperm particles after staining with C_11_-BODIPY^581/591^ and PI. **Figure S6.** Flow cytometric dot-plot distributions of spermatozoa and non-sperm particles after staining with AO.

## Data Availability

The datasets used during the current study are available from the corresponding author on reasonable request.

## Abbreviations

ANISO: anisotropy
ALH: Amplitude of lateral head displacement
AO: Acridine orange dye
ART: Assisted reproductive techniques
BCF: Beat cross frequency
CASA: Computer assisted semen analysis
CLC: Cholesterol loaded cyclodextrin
DPH: 1,6-diphenyl-1,3,5-hexatriene
GP: Generalized polarization
HBCD: 2-hydroxypropyl-β-cyclodextrin
HMMP: High mitochondrial membrane potential
LIN: Linearity
MOT: Motility
PI: Propidium iodide
PMOT: Progressive motility
RAP: The percentage of rapid spermatozoa
STR: Straightness
TFE: Tris-citric acid–fructose–egg yolk extender VAP: Average path velocity
VCL: Curvilinear velocity
VSL: Straight line velocity

## References

[1] Nizański W, Dubiel A, Bielas W, Dejneka GJ (2001). Effects of three cryopreservation methods and two semen extenders on the quality of dog semen after thawing. J Reprod Fertil Suppl..

[2] Hori T, Yoshikuni R, Kobayashi M, Kawakami E (2014). Effects of storage temperature and semen extender on stored canine semen. J Vet Med Sci..

[3] Grandhaye J, Partyka A, Ligocka Z (2020). Metformin improves quality of post-thaw canine semen. Animals (Basel).

[4] Lechner D, Aurich J, Schäfer-Somi S, Herbel J, Aurich C (2021). Combined cryopreservation of canine ejaculates collected at a one-hour interval increases semen doses for artificial insemination without negative effects on post-thaw sperm characteristics. Reprod Domest Anim..

[5] Domain G, Ali Hassan H, Wydooghe E (2022). Influence of single layer centrifugation with canicoll on semen freezability in dogs. Animals (Basel)..

[6] Pinto CR, Paccamonti DL, Eilts BE (1999). Fertility in bitches artificially inseminated with extended, chilled semen. Theriogenology..

[7] Bailey JL, Bilodeau JF, Cormier N (2000). Semen cryopreservation in domestic animals: a damaging and capacitating phenomenon. J Androl..

[8] Moore AI, Squires EL, Graham JK (2005). Adding cholesterol to the stallion sperm plasma membrane improves cryosurvival. Cryobiology..

[9] Raffy S, Teissié J (1999). Control of lipid membrane stability by cholesterol content. Biophys J..

[10] López C, de Vries A, Marrink S (2013). Computational microscopy of cyclodextrin mediated cholesterol extraction from lipid model membranes. Sci Rep.

[11] Mocé E, Blanch E, Tomás C, Graham JK (2010). Use of cholesterol in sperm cryopreservation: present moment and perspectives to future. Reprod Domest Anim..

[12] Lone SA (2018). Possible mechanisms of cholesterol-loaded cyclodextrin action on sperm during cryopreservation. Anim Reprod Sci..

[13] Purdy PH, Graham JK (2004). Effect of cholesterol-loaded cyclodextrin on the cryosurvival of bull sperm. Cryobiology..

[14] Blommaert D, Franck T, Donnay I, Lejeune JP, Detilleux J, Serteyn D (2016). Substitution of egg yolk by a cyclodextrin-cholesterol complex allows a reduction of the glycerol concentration into the freezing medium of equine sperm. Cryobiology..

[15] Visconti PE, Galantino-Homer H, Ning X, Moore GD, Valenzuela JP, Jorgez CJ, Alvarez JG, Kopf GS (1999). Cholesterol efflux-mediated signal transduction inmammalian sperm. Beta-cyclodextrins initiate transmem-brane signaling leading to an increase in protein tyrosinephosphorylation and capacitation.J. Biol. Chem..

[16] Williams RO, Mahaguna V, Sriwongjanya M (1998). Characterization of an inclusion complex of cholesterol and hydroxypropyl-beta-cyclodextrin. Eur J Pharm Biopharm..

[17] Szente L, Fenyvesi É (2017). Cyclodextrin-Lipid Complexes: Cavity Size Matters. Struct Chem.

[18] Dos Santos AG, Bayiha JC, Dufour G (2017). Changes in membrane biophysical properties induced by the Budesonide/Hydroxypropyl-β-cyclodextrin complex. Biochim Biophys Acta Biomembr.

[19] Blesbois E, Grasseau I, Seigneurin F (2005). Membrane fluidity and the ability of domestic bird spermatozoa to survive cryopreservation. Reproduction..

[20] Konyali C, Tomás C, Blanch E, Gómez EA, Graham JK, Mocé E (2013). Optimizing conditions for treating goat semen with cholesterol-loaded cyclodextrins prior to freezing to improve cryosurvival. Cryobiology..

[21] Amorim EA, Graham JK, Spizziri B, Meyers M, Torres CA (2009). Effect of cholesterol or cholesteryl conjugates on the cryosurvival of bull sperm. Cryobiology..

[22] Mocé E, Purdy PH, Graham JK (2010). Treating ram sperm with cholesterol-loaded cyclodextrins improves cryosurvival. Anim Reprod Sci..

[23] Naseer Z, Ahmad E, Aksoy M (2015). Protective effect of cholesterol-loaded cyclodextrin pretreatment against hydrogen peroxide induced oxidative damage in ram sperm. Cryobiology..

[24] Lee YS, Lee S, Lee SH, Yang BK, Park CK (2015). Effect of cholesterol-loaded-cyclodextrin on sperm viability and acrosome reaction in boar semen cryopreservation. Anim Reprod Sci..

[25] Khan J, Tahir MZ, Khalid A, Sattar A, Ahmad N (2017). Effect of cholesterol-loaded cyclodextrins on cryosurvival of dog spermatozoa. Reprod Domest Anim..

[26] Rijsselaere T, Maes D, Hoflack G, de Kruif A, Van Soom A (2007). Effect of body weight, age and breeding history on canine sperm quality parameters measured by the Hamilton-Thorne analyser. Reprod Domest Anim..

[27] Schäfer-Somi S, Palme N (2016). Seminal Plasma Characteristics and Expression of ATP-binding Cassette Transporter A1 (ABCA1) in Canine Spermatozoa from Ejaculates with Good and Bad Freezability. Reprod Domest Anim..

[28] Eilts BE (2005). Theoretical aspects of canine cryopreserved semen evaluation. Theriogenology.

[29] Linde-Forsberg C, Ström Holst B, Govette G (1999). Comparison of fertility data from vaginal vs intrauterine insemination of frozen-thawed dog semen: a retrospective study. Theriogenology.

[30] Moraes EA, Matos WC, Graham JK, Ferrari WD (2015). Cholestanol-loaded-cyclodextrin improves the quality of stallion spermatozoa after cryopreservation. Anim Reprod Sci..

[31] Neagu VR, García BM, Sandoval CS (2010). Freezing dog semen in presence of the antioxidant butylated hydroxytoluene improves postthaw sperm membrane integrity. Theriogenology..

[32] Rota A, Ström B, Linde-Forsberg C, Rodriguez-Martinez H (1997). Effects of equex STM paste on viability of frozen-thawed dog spermatozoa during in vitro incubation at 38 degrees C. Theriogenology..

[33] Rota A, Iguer-Ouada M, Verstegen J, Linde-Forsberg C (1999). Fertility after vaginal or uterine deposition of dog semen frozen in a tris extender with or without Equex STM paste. Theriogenology..

[34] Peña A, Linde-Forsberg CB (2000). Effects of spermatozoal concentration and post-thaw dilution rate on survival after thawing of dog spermatozoa. Theriogenology..

[35] Wojtusik J, Pennington P, Songsasen N, Padilla LR, Citino SB, Pukazhenthi BS (2016). Pretreatment of Addra gazelle (Nanger dama ruficollis) spermatozoa with cholesterol-loaded cyclodextrins improves cryosurvival. Cryobiology..

[36] Yadav HP, Kumar A, Shah N (2017). Effect of cholesterol loaded cyclodextrin supplementation on tyrosine phosphorylation and apoptosis like changes in frozen thawed Hariana bull spermatozoa. Theriogenology..

[37] Galantino-Homer HL, Zeng WX, Megee SO, Dallmeyer M, Voelkl D, Dobrinski I (2006). Effects of 2-hydroxypropyl-beta-cyclodextrin and cholesterol on porcine sperm viability and capacitation status following cold shock or incubation. Mol Reprod Dev..

[38] Holt WV (2000). Fundamental aspects of sperm cryobiology: the importance of species and individual differences. Theriogenology..

[39] Watson PF, Morris GJ, Clarke A (1981). The Effect of Cold Shock on Sperm Cell Membranes. The Effect of Low Temperature on Biological Membranes.

[40] Parks JE, Lynch DV (1992). Lipid composition and thermotropic phase behavior of boar, bull, stallion, and rooster sperm membranes. Cryobiology..

[41] Brinsko SP, Love CC, Bauer JE, Macpherson ML, Varner DD (2007). Cholesterol-to-phospholipid ratio in whole sperm and seminal plasma from fertile stallions and stallions with unexplained subfertility. Anim Reprod Sci..

[42] Beer-Ljubić B, Aladrović J, Marenjak TS, Laskaj R, Majić-Balić I, Milinković-Tur S (2009). Cholesterol concentration in seminal plasma as a predictive tool for quality semen evaluation. Theriogenology..

[43] Argov N, Sklan D, Zeron Y, Roth Z (2007). Association between seasonal changes in fatty-acid composition, expression of VLDL receptor and bovine sperm quality. Theriogenology..

[44] Inanc ME, Tekin K, Olgac KT (2018). Effect of cholesterol loaded cyclodextrin on semen cryopreservation of Aksaray Malakli shepherd dogs of different ages. Anim Reprod Sci..

[45] Esteves SC, Sharma RK, Thomas AJ, Agarwal A (2007). Evaluation of acrosomal status and sperm viability in fresh and cryopreserved specimens by the use of fluorescent peanut agglutinin lectin in conjunction with hypo-osmotic swelling test. Int Braz J Urol..

[46] Tomás C, Blanch E, Hernández M (2011). Treating boar sperm with cholesterol-loaded cyclodextrins widens the sperm osmotic tolerance limits and enhances the in vitro sperm fertilising ability. Anim Reprod Sci..

[47] Aitken RJ (1995). Free radicals, lipid peroxidation and sperm function. Reprod Fertil Dev..

[48] Sicherle CC, de Souza FF, Freitas-Dell’Aqua CP, Mothé GB, Padovani CR, Papa FO, Lopes MD (2020). Effects of the cryopreservation process on dog sperm integrity. Animal reproduction.

[49] Lone SA, Prasad J, Ghosh SK, Das GK, Kumar N, Balamurugan B, Katiyar R, Verma MR (2016). Effect of cholesterol loaded cyclodextrin (CLC) on lipid peroxidation and reactive oxygen species levels during cryopreservation of buffalo (Bubalus bubalis) spermatozoa. Asian Pac J Reprod.

[50] Lewis SEM, Aitken RJ (2005). DNA damage to spermatozoa has impacts on fertilization and pregnancy. Cell Tissue Res.

[51] Martinez-Pastor F, Johannisson A, Gil J (2004). Use of chromatin stability assay, mitochondrial stain JC-1, and fluorometric assessment of plasma membrane to evaluate frozen-thawed ram semen. Anim Reprod Sci..

[52] Nizański W (2006). Intravaginal insemination of bitches with fresh and frozen-thawed semen with addition of prostatic fluid: use of an infusion pipette and the Osiris catheter. Theriogenology..

[53] Stone BA, Alex A, Werlin LB, Marrs RP (2013). Age thresholds for changes in semen parameters in men. Fertil Steril..

[54] Rota A, Tesi M, Di Petta G, Sabatini C, Vannozzi I (2016). A retrospective study on the relationships between semen quality, dogs’ ageing and fertility. 8th International Symposium on Canine and Feline Reproduction ISCFR.

[55] Partyka A, Nizański W, Łukaszewicz E (2010). Evaluation of fresh and frozen-thawed fowl semen by flow cytometry. Theriogenology..

[56] Partyka A, Niżański W, Bajzert J, Łukaszewicz E, Ochota M (2013). The effect of cysteine and superoxide dismutase on the quality of post-thawed chicken sperm. Cryobiology..

[57] Peña FJ, Johannisson A, Wallgren M, Rodriguez Martinez H (2004). Antioxidant supplementation of boar spermatozoa from different fractions of the ejaculate improves cryopreservation: changes in sperm membrane lipid architecture. Zygote..

[58] Partyka A, Lukaszewicz E, Niżański W, Twardoń J (2011). Detection of lipid peroxidation in frozen-thawed avian spermatozoa using C(11)-BODIPY(581/591). Theriogenology..

[59] Parasassi T, Loiero M, Raimondi M, Ravagnan G, Gratton E (1993). Absence of lipid gel-phase domains in seven mammalian cell lines and in four primary cell types. Biochim Biophys Acta..

[60] Team RC R (2023). A language and environment for statistical computing.

[61] Kassambara A (2019). Comparing groups: Numerical variables.

[62] Revelle WR (2017). psych: Procedures for personality and psychological research.

[63] Bender R, Lange S (2001). Adjusting for multiple testing—when and how?. J Clin Epidemiol.

[64] Dray S, Dufour AB (2007). The ade4 package: implementing the duality diagram for ecologists. J Stat Softw.

[65] Bougeard S, Dray S (2018). Supervised Multiblock Analysis in R with the ade4 Package. J Stat Softw.

[66] Chessel D, Dufour A, Thioulouse J (2004). The ade4 Package – I: One-Table Methods. R News.

[67] Dray S, Dufour A, Chessel D (2007). The ade4 Package – II: Two-Table and K-Table Methods. R News.

[68] Thioulouse J, Dray S, Dufour A, Siberchicot A, Jombart T, Pavoine S. Multivariate Analysis of Ecological Data with ade4_. Springer. 2018, 10.1007/978-1-4939-8850-1

[69] Kassambara A, Mundt F. Factoextra: extract and visualize the results of multivariate data analyses. R Package Version 1.0.7. 2020. https://CRAN.R-project.org/package=factoextra.

