# Supplementary material for: Addition of low concentration of cholesterol-loaded cyclodextrin (CLC) has a positive effect on cryopreserved canine spermatozoa evaluated by andrological and biophysical methods
Source: BMC Vet Res. 2024 Jan 3;20:7. doi: 10.1186/s12917-023-03851-6 (PMC10763487; doi:10.1186/s12917-023-03851-6)
Supplement: Supplementary file 1 — Additional file 1: Figure S1. Flow cytometric dot-plot distributions of spermatozoa and non-sperm particles after staining with SYBR-14 and PI. Figure S2. Flow cytometric dot-plot distributions of spermatozoa and non-sperm particles after staining with PNA and PI. Figure S3. Flow cytometric dot-plot distributions of spermatozoa and non-sperm particles after staining with JC-1 and PI. Figure S4. Flow cytometric dot-plot distributions of spermatozoa and non-sperm particles after staining with YO-PRO-1 and M540. Figure S5. Flow cytometric dot-plot distributions of spermatozoa and non-sperm particles after staining with C11-BODIPY581/591 and PI. Figure S6. Flow cytometric dot-plot distributions of spermatozoa and non-sperm particles after staining with AO. [file 12917_2023_3851_MOESM1_ESM.docx]

Supplementary material


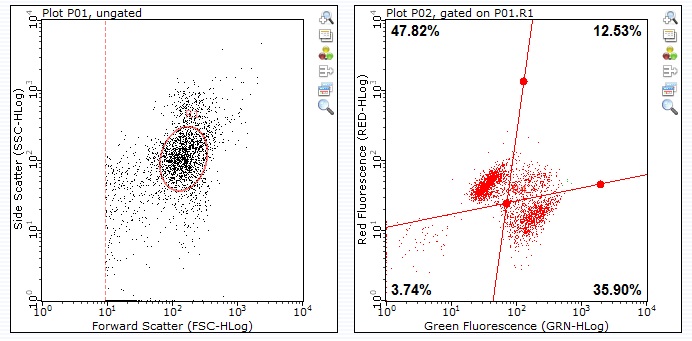


Figure S1 Flow cytometric dot-plot distributions of spermatozoa and non-sperm particles after staining with SYBR-14 and PI.


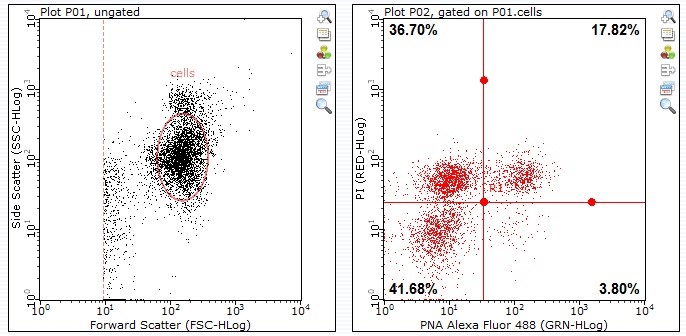


Figure S2 Flow cytometric dot-plot distributions of spermatozoa and non-sperm particles after staining with PNA and PI.


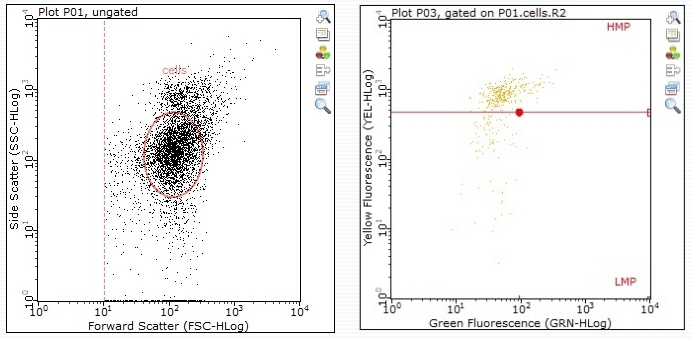


Figure S3 Flow cytometric dot-plot distributions of spermatozoa and non-sperm particles after staining with JC-1 and PI.


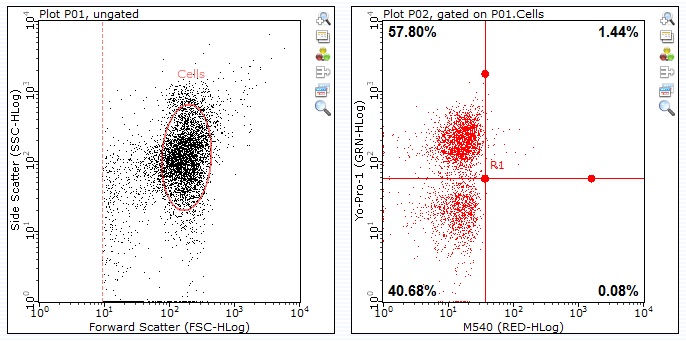


Figure S4 Flow cytometric dot-plot distributions of spermatozoa and non-sperm particles after staining with YO-PRO-1 and M540.


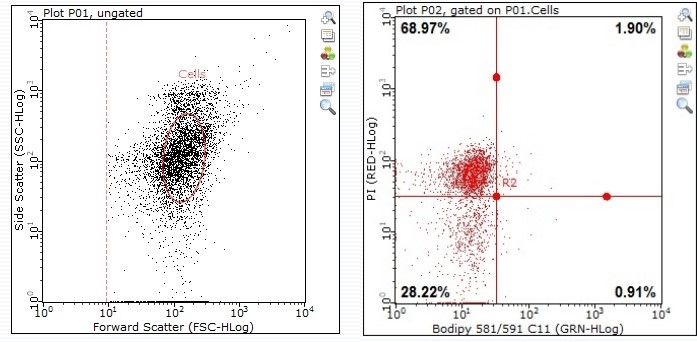


Figure S5 Flow cytometric dot-plot distributions of spermatozoa and non-sperm particles after staining with C_11_-BODIPY^581/591^ and PI.


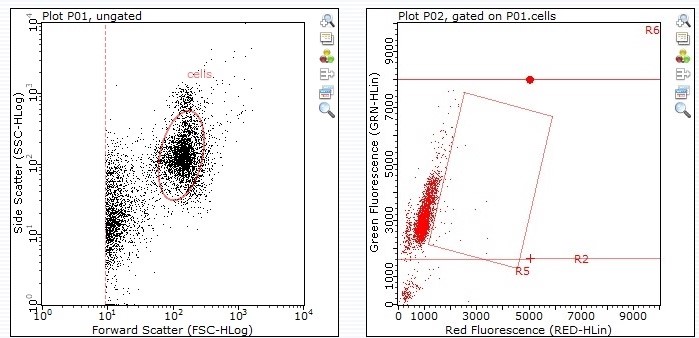


Figure S6 Flow cytometric dot-plot distributions of spermatozoa and non-sperm particles after staining with AO.
